# Post-Operative Auto-Transfusion in Total Hip or Knee Arthroplasty: A Meta-Analysis of Randomized Controlled Trials

**DOI:** 10.1371/journal.pone.0055073

**Published:** 2013-01-25

**Authors:** Zhao Haien, Jiang Yong, Ma Baoan, Guo Mingjun, Fan Qingyu

**Affiliations:** 1 Department of Orthopaedic Surgery, Tangdu Hospital, The Fourth Military Medical University, Xi’an, China; 2 Department of Orthopaedic Surgery, Hong Hui Hospital, Xi'an Jiaotong University College of Medicine, Xi’an, China; Cardiff University, United Kingdom

## Abstract

**Background:**

Total hip or knee arthroplasty is an elective procedure that is usually accompanied by substantial blood loss, which may lead to acute anemia. As a result, almost half of total joint arthroplasty patients receive allogeneic blood transfusions (ABT). Many studies have shown that post-operative auto-transfusion (PAT) significantly reduces the need for ABT, but other studies have questioned the efficacy of this method.

**Methods:**

The protocol for this trial and supporting CONSORT checklist are available as supporting information; see Checklist S1. To evaluate the efficacy of PAT, we conducted a Cochrane systematic review that combined all available data from randomized controlled trials. Data from the six included trials were pooled for analysis. We then calculated relative risks with 95% confidence intervals (CIs) for dichotomous outcomes and mean differences with 95% CIs for continuous outcomes.

**Findings and Conclusion:**

To our knowledge, this is the first meta-analysis to compare the clinical results between PAT and a control in joint replacement patients. This meta-analysis has proven that the use of a PAT reinfusion system reduced significantly the demand for ABT, the number of patients who require ABT and the cost of hospitalization after total knee and hip arthroplasty. This study, together with other previously published data, suggests that PAT drains are beneficial. Larger, sufficiently powered studies are necessary to evaluate the presumed reduction in the incidence of infection as well as DVT after joint arthroplasty with the use of PAT.

## Introduction

Total hip or knee arthroplasty is an elective procedure that is usually accompanied by substantial blood loss, which may lead to acute anemia. As a result, almost half of total joint arthroplasty patients receive allogeneic blood transfusions (ABT) to prevent postoperative anemia [1]–[4]. However, ABT is an increasingly expensive and scarce resource, and ABT itself is not a risk-free therapeutic method. ABT can lead to the transmission of infectious diseases such as HIV, hepatitis, cytomegalovirus, Epstein-Barr virus, syphilis, and malaria as well as transfusion-related acute lung injury, hemolytic reactions, fluid overload, and an increased rate of postoperative infection [5], [6].

An increased awareness of the greater incidence of postoperative complications and other potential hazards of ABT has prompted a review of transfusion practices and a search for blood conservation measures, such as the correction of peri-operative anemia (e.g., intravenous iron, recombinant human erythropoietin), the use of pharmacological agents to reduce peri-operative blood loss (eg, aprotinin, tranexamic acid), and the use of different measures of autologous blood transfusion [5]–[7]. Autologous blood transfusion includes pre-operative donation, intra-operative auto-transfusion (IAT), and post-operative auto-transfusion (PAT) [8]. In PAT, the post-operative blood drainage collected in drains is salvaged and returned. Many studies have shown that PAT significantly reduced the need for ABT [9]–[11], but other studies have questioned the efficacy of this method [12], [13] or found that PAT after arthroplasty had a limited effect in terms of blood conservation [3], [4].

The main objective of this study was to compare the clinical results of post-operative auto-transfusion (PAT) with the results of a control group that received standardized drainage (if necessary, patients were given allogeneic blood transfusion) following total hip or knee arthroplasty. To evaluate the clinical efficacy of PAT, we conducted a meta-analysis that combined all of the available data from randomized controlled trials (RCTs).

## Materials and Methods

### Literature Search

We searched for the published results of relevant trials in the Cochrane Library (Issue 2, 2011), PubMed (January 1995 to February 2011), Ovid (January 1995 to February 2011), ScienceDirect Online (January 1995 to February 2011), ISI Web of Knowledge (January 1995 to February 2011), several orthopedic journals, transfusion journals, and conference proceedings. When necessary, the authors of the articles were contacted for original information. The search terms used included “total hip replacement”, “total hip arthroplasty”, “total knee replacement”, “total knee arthroplasty”, “total joint arthroplasty”, “autotransfusion”, “autologous transfusion”, “post-operative blood salvage”, “post-operative transfusion” and “randomized controlled trials”.

### Inclusion and Exclusion Criteria

We retrieved all randomized controlled trials (RCTs) that compared the PAT system group with the control group in patients undergoing total knee or hip arthroplasty. Properly randomized trials were eligible for inclusion if (i) the results included at least one of the outcome measures; (ii) at least one of the data points was presented as the mean ± SD; (iii) all data were shown as the medians and/or ranges, and we obtained the original information by contacting the corresponding author; (iv) there was no consideration of primary replacement or revision replacement of the joint.

Patients were excluded from the trials if (i) they had received any other blood-saving strategy including preoperative autologous blood donation, erythropoietin treatment, iron supplementation; (ii) all data were shown as the medians and/or ranges, and it was not possible to obtain the original information by contacting the author.

The data were extracted by 2 reviewers independently to ensure accuracy. In cases of disagreement, a consensus was reached by discussion. Study quality was evaluated according to the method for RCTs described in the Cochrane Reviewer’s Handbook 5.0 [14].

### Outcome Measures

The primary outcome measures assessed in this analysis include the number of patients requiring at least one unit of allogeneic RBCs (patients requiring homologous blood transfusions).

The secondary outcome measures include the ABT index (units of RBC transfused per patient), the total volume of blood loss following surgery, postoperative hemoglobin (Hb) level, transfusion reactions, infections, deep vein thrombosis (DVT), and the length of the hospital stay (LOHS).

### Statistical Analysis

The data were pooled using REVMAN 5.0 software (The Nordic Cochrane Centre, Copenhagen, Denmark). For each study, we calculated relative risks (RRs) with 95% confidence intervals (CIs) for dichotomous data and mean differences (MDs) with 95% CIs for continuous data. Where appropriate, we pooled the results of comparable groups of trials using the fixed-effect (Mantel-Haenszel test) and random-effect (DerSimonian-Laird method) models. A random-effect model was used when significant heterogeneity was detected between studies (P<0.10; I^2^>50%). Otherwise, a fixed-effect model was used.

## Results

### Literature Search

**Figure 1:**
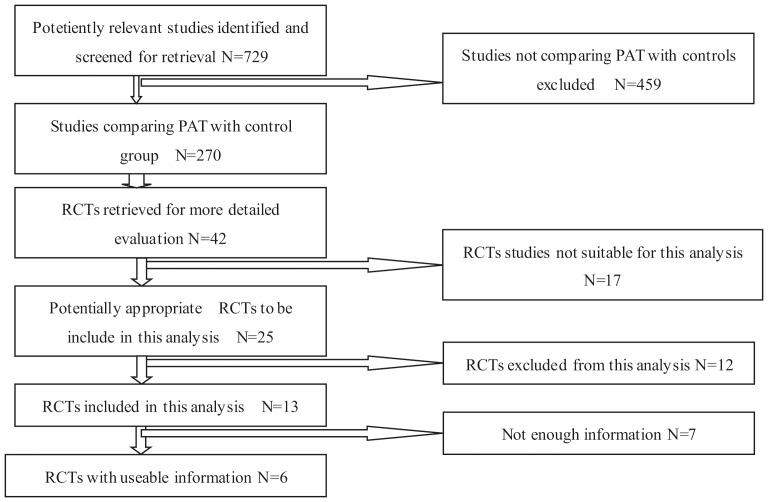
The QUOROM diagram for review. RCT, randomized controlled trial.

The preliminary literature search yielded 729 potentially relevant articles. Most of these studies, however, were non-randomized cohort studies, retrospective studies, biomechanical studies, case reports, or other forms of investigation that did not fit our inclusion criteria. Finally, six studies were identified as eligible for data extraction and meta-analysis [3], [12], [13], [15], [18], [19]. These six studies were adequately randomized studies of RCTs in which consensus was reached by discussion (Figure 1). The allocation concealments of all six eligible studies were unclear. None of the studies included adequate blinding procedures. In total, 829 patients from these six trials were included in our analysis; 469 of these patients received post-operative auto-transfusion (PAT) and 360 patients served as the control group. The quality of the trials included in this meta-analysis is presented in Table 1.

**Table 1:** Quality evaluation of included RCTs.

Inclusion study	Design	Number		Random	Concealment	Blinding	Follow-up	Grade
		PAT	CG					
A.Amin 2008	RCT	92	86	Y	Y	UC	Y	B
Adrianus 2007	RCT	80	80	Y	Y	UC	Y	B
C.soosman 2006	RCT	47	22	Y	Y	UC	Y	B
Konstantino2010	RCT	163	85	Y	UC	UC	Y	C
Newman 1997	RCT	35	35	Y	UC	UC	Y	B
T.abuzakuk 2007	RCT	52	52	Y	UC	UC	Y	B

RCTs: Randomized controlled trials; PAT: Post-operative autotransfusion group; CG: Control group; Y: Yes; UC: Unclear.

### Outcome Measures

**Figure 2:**
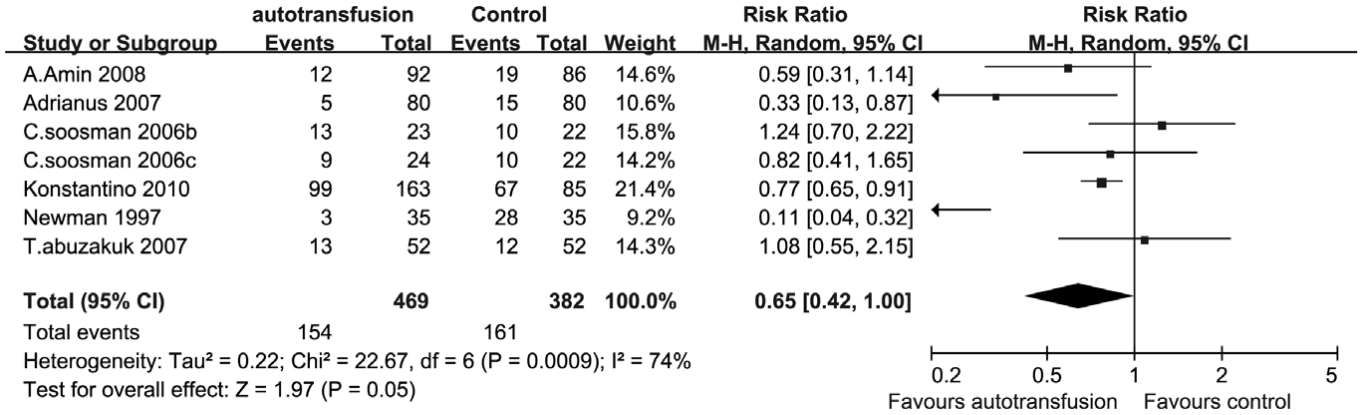
The RRs and 95% CIs for the number of patients requiring at least one unit of ABT among patients treated with vs. without PAT. It indicated that RRs of patients requiring ABT in the PAT group was significantly lower than that in the control group.

All of the six RCTs reported the number of patients requiring at least one unit of allogeneic RBCs following the arthroplasty surgery. So we included the six RCTs as the data of the meta-analysis in figure 2. In one of the studies (Soosman 2006), patients were randomized to three groups: group A as a control group, and group B and group C as two different re-infusion system groups. In our analysis, as a results, Soosman’s study was divided into two studies, as Soosman 2006b and Soosman 2006c. Because significant heterogeneity was detected between the studies (P = 0.0009; I^2^ = 74%), a random-effect model was used. The pooled result indicated a significant difference between treatment groups in the numbers of patients requiring at least one unit of allogeneic RBCs (RRs, 0.65; 95% CIs, 0.42 to 1.00; P = 0.05) (Figure 2).

**Figure 3:**
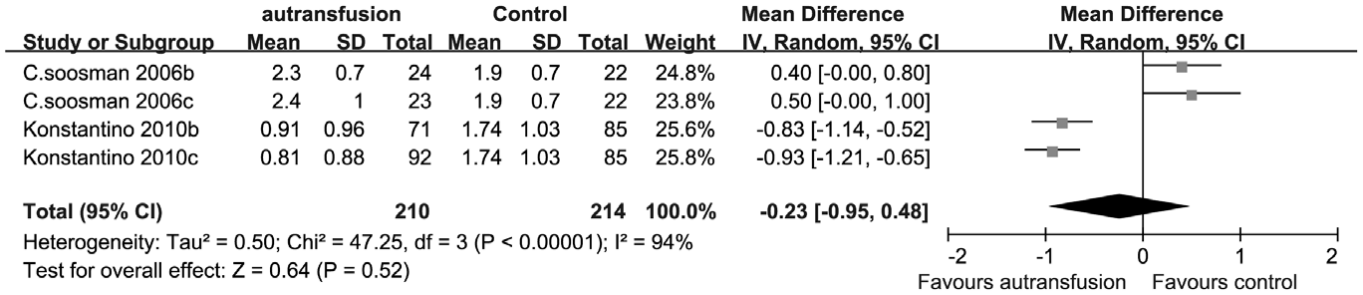
The MDs and 95% CIs for units of RBC per transfused patient among patients treated with vs. without PAT. The pooled result demonstrated that there was no significant difference of the ABT index between the PAT group and the control group.

Two RCTs (Soosman 2006 and Konstantino 2010) was included in the meta-analysis of ABT index. In these two studies, patients were randomized to three groups: group A as a control group, and group B and group C as two different re-infusion system groups. As a results, Soosman’s study and Konstantino’s study was divided into two studies, as Soosman 2006b and Soosman 2006c, Konstantino 2010b and Konstantino 2010c, respectively. It demonstrated that there was no heterogeneity between subtotal (P = 0.99; I^2^ = 0%) and we could pooled the two autotransfusion techniques in a meta-analysis. A random-effect model was used because significant heterogeneity was detected among the two studies of autotransfusion devices (P<0.00001; I^2^ = 94%). The pooled result of RCTs indicated that the ABT index differences between the PAT group and the control group were not statistically significant (MDs, −0.23; 95% CIs, −0.95 to 0.48; P = 0.52) (Figure 3).

**Figure 4:**
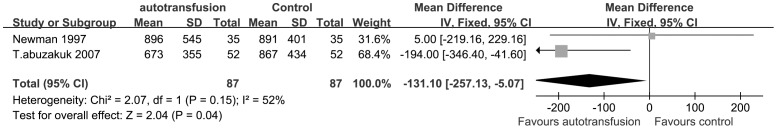
The MDs and 95% CIs for the volume of blood loss among patients treated with vs. without PAT. The result indicated significantly less volume of blood loss in the PAT group than in the control group.

Among six of the trials, three studies reported the volume of the blood loss following surgery compared the total amount of blood loss in the PAT group with a control group, including both the intra-operative and post-operative blood loss. One of the five studies (Soosman 2006) provided the volume as a median and range rather than as the mean ± SD. Although we tried to contact the corresponding author, we could not obtain the original information. Thus, two studies were included in our comparison of the volume of blood loss. We pooled the two RCTs data in Figure 4. Because no significant heterogeneity was observed among these studies (P = 0.15), a fixed-effect model was used. The pooled results indicated less volume of blood loss in the autotransfusion group than in the control group (MDs, −131.10; 95% CIs, −257.13 to −5.07; P = 0.04) (Figure 4). The result showed differences between the groups were statistically significant.

**Figure 5:**
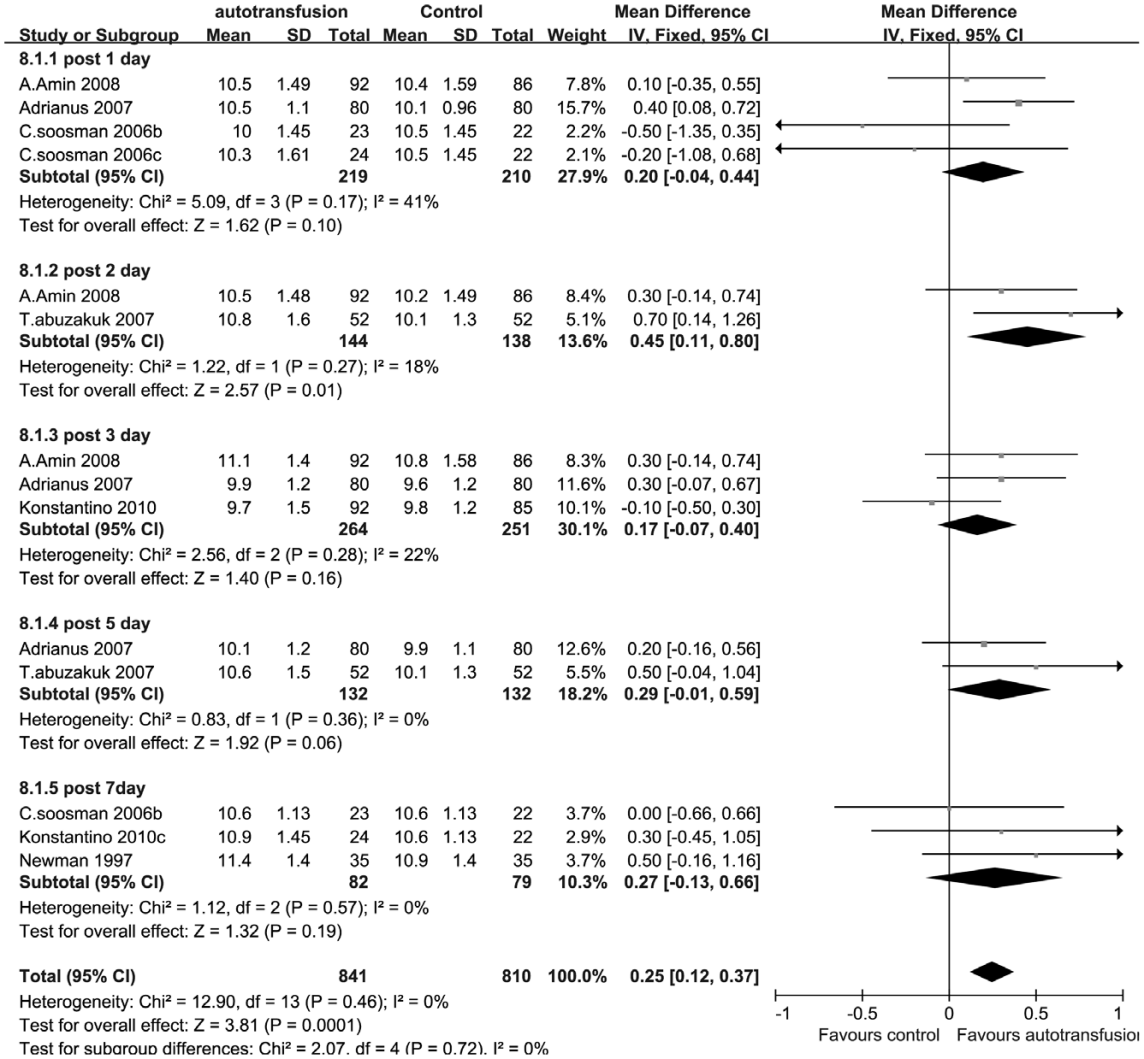
The MDs and 95% CIs for the post-operative Hb level among patients treated with vs. without PAT. It demonstrated that post-operative Hb was significantly higher in the PAT group than in the control group.

The post-operation Hb level data were divided into 5 subgroups (1, 2, 3, 5, and 7 days post-operation) based on the post-operative time at which the Hb level data were acquired. Because no significant heterogeneity was observed among the subgroups (P = 0.46; I^2^ = 0%) in this comparison, a fixed-effect model was employed. The pooled results of RCTs revealed a significant difference in the post-operative Hb level between the PAT group and the control group. The results demonstrated a higher level of post-operative Hb in the auto-transfusion group than in the control group (MDs, 0.25; 95% CIs, 0.12 to 0.37; P = 0.0001) (Figure 5).

**Figure 6:**
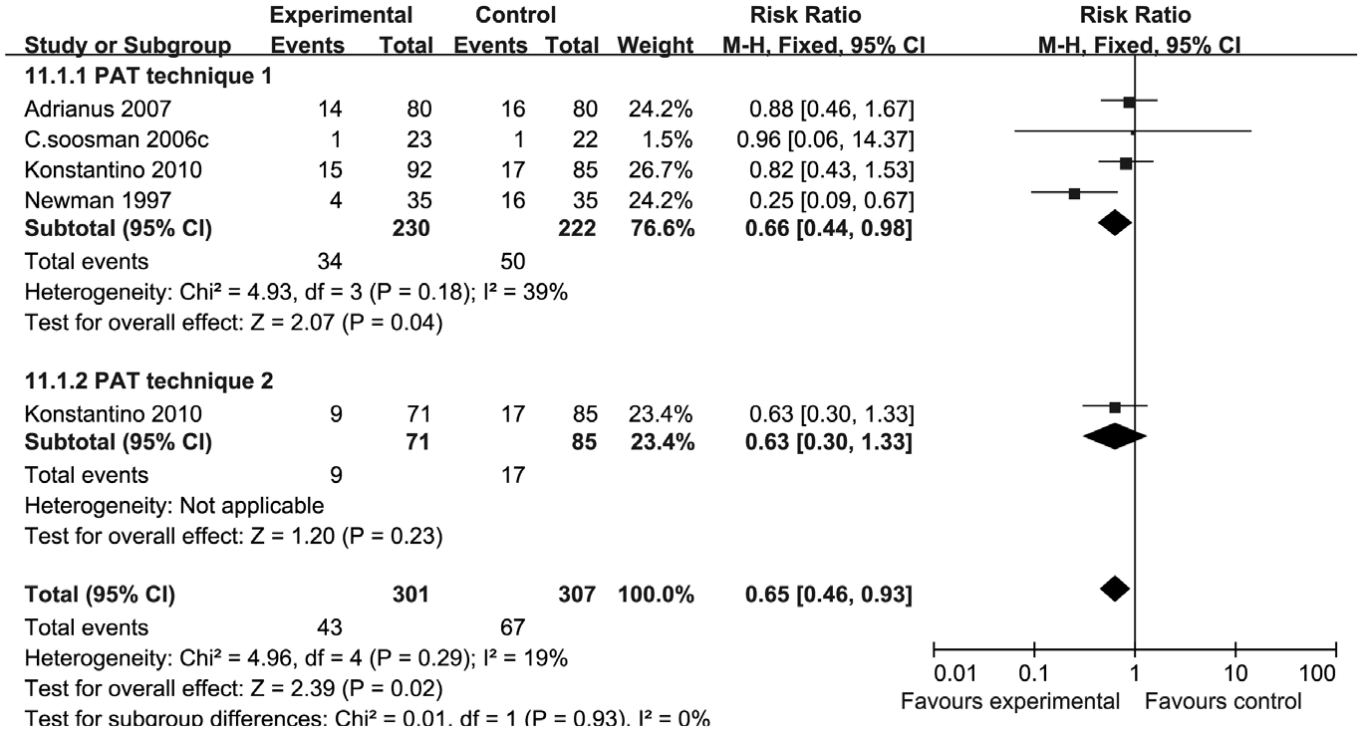
The RRs and 95% CIs for the incidence of febrile reaction among patients treated with vs. without PAT. It indicated that the febrile reaction rate in the PAT group decreased significantly compared with the control group.

Four RCTs trials reported febrile reactions among patients who received PAT versus ABT. Heterogeneity between the two treatment groups was not statistically significant (P = 0.29; I^2^ = 19%), so we employed a fixed-effect model to study the febrile reaction. The pooled results revealed a significant difference in the febrile reaction rate between the transfusion treatment groups (RRs, 0.65; 95% CIs, 0.46 to 0.93; P = 0.02) (Figure 6).

**Figure 7:**
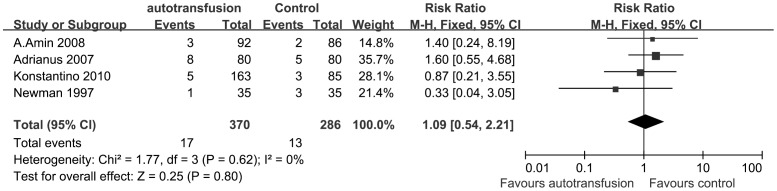
The RRs and 95% CIs for the incidence of infection among patients treated with vs. without PAT. The result revealed no significant difference between the treatment groups.

Four RCTs was included in the study of the infection rate. We pooled the results of the RCTs in Figure 7. Figure 7 shows the difference in the infection rate between PAT patients and ABT patients after total joint arthroplasty surgery. We found no significant heterogeneity between studies (P = 0.62; I^2^ = 0%) and thus compared the data with a fixed-effect model. The pooled result revealed no significant difference between the treatment groups (RRs, 1.09; 95% CIs, 0.54 to 2.21; P = 0.80).

**Figure 8:**
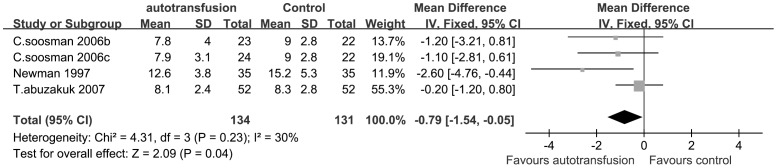
The MDs and 95% CIs for the length of hospital stay among patients treated with vs. without PAT. It demonstrated LOHS in the PAT patients was shorter than patients in the control group after surgery.

Three RCTs were included in the study of LOHS and we pooled these RCTs in the study of LOHS in Figure 8. With no significant heterogeneity among the data compared in LOHS (P = 0.23; I^2^ = 30%; fixed-effect analysis), the pooled result revealed a significant difference in LOHS between the treatment groups (MDs, −0.79; 95% CIs, −1.54 to −0.05; P = 0.04) (Figure 8). It demonstrated LOHS in the PAT patients was shorter than patients in the control group after surgery. Two RCTs studies reported the incidence of DVT and revealed a lack of significant differences between the PAT group and the control group (RRs, 0.38; 95% CIs, 0.06 to 2.51; P = 0.31) (figure not shown).

## Discussion

Total knee or hip arthroplasty is usually accompanied by substantial blood loss and regularly results in a postoperative requirement for blood transfusion. Because of the disadvantages of allogeneic blood transfusion (ABT) such as the risk of transfusion-associated infections, incompatibility-related transfusion fatalities or immuno-modulatory effects, a continuing effort to reduce ABT is important. Thus, the reinfusion of drained blood via a postoperative wound drainage and reinfusion system and the need for ABT was evaluated.

As mentioned above, autologous blood transfusion includes pre-operative blood donation, intra-operative auto-transfusion (IAT), and PAT. In pre-operative blood donation, the patient’s own blood is collected a few weeks before the operation and transfused post-operatively as needed. In IAT, the patient’s blood is collected from the operating field and reinfused. In PAT, the post-operative blood drainage collected in drains is salvaged and returned [20].

Recent techniques have facilitated autologous blood transfusion, and the concept of reinfusing blood collected in the drain following total joint arthroplasty is becoming increasingly attractive to many surgeons. However, PAT following surgery continues to be a controversial topic in the orthopedic literature, and many studies have reported considerable doubt with respect to its benefits [21], [22].

In orthopedic surgery, the auto-transfusion of washed (RRs, 0.33; 95% CIs, 0.23 to 0.47) or unwashed cells (RRs, 0.30; 95% CIs, 0.21 to 0.43) decreased the frequency of exposure to ABT to a similar degree when compared with a control. Some studies had shown a significant reduction in the rate of ABT following the use of PAT systems [4], [5], [23]–[26]. Recently, Strümper et al. reported a significant reduction in the rate of ABT among patients treated with PAT [4], [15]. Sinha et al. found that ABT was reduced by 80% in an autologous reinfusion group as compared with the standard suction group (RRs, 0.18; 95% CIs, 0.10 to 0.35; P<0.00001) [16]. Newman et al found that 3 of 35 patients in the PAT drain group required ABT, compared with 28 of 35 patients in the control group (RRs, 0.11; 95% CIs, 0.04 to 0.32; P<0.0001) [19]. A recent meta-analysis of RCTs found that PAT reduced the probability of receiving ABT for orthopedic surgery (RRs, 0.35, 95% CIs, 0.24 to 0.52) [27]. del Trujillo et al. found that PAT could decrease the exposure to ABT by 69% and could significantly reduce the units of allogeneic RBC required (MDs, −1.59; 95% CIs, −1.85 to −1.33; P<0.00001) in patients undergoing cemented or uncemented total arthroplasty [17]. The ABT rate has also been found to be significantly lower in patients receiving PAT after total knee arthroplasty [4], [27], [28]. Newman et al. also believed that PAT alone was safe and effective after unilateral total knee arthroplasty, as it is the least troublesome and the least expensive of the available methods of autologous transfusion [19]. Several studies previously showed that unwashed and filtered blood was of satisfactory quality and was safe to reinfuse [19], [29].

Contrary to the above-mentioned results in which the use of a PAT system was shown to be advantageous, some authors have suggested insufficient efficiency for PAT [21], [30]. Buzakuk et al. found that PAT transfusion failed to reduce the need for postoperative ABT following primary total knee arthroplasty (RRs, 1.08; 95% CIs, 0.55 to 2.15; P = 0.82) [12]. Rollo reported no reduction in allogeneic RBC transfusions in total hip arthroplasty patients [31]. Ritter et al. and others failed to show a PAT system could reduce the need for ABT in patients scheduled for total hip and knee replacements [32]. Moreover, recent studies found that PAT after hip arthroplasty had a limited blood-conservation effect [3], [4], [33].

The pooled results of our study demonstrated significant decreases in the rate of homologous blood transfusion compared the PAT group with the control group (RRs, 0.65; 95% CIs, 0.42 to 1.00; P = 0.05) (Figure 2). This meta-analysis identified statistically significant reduction in the number of patients receiving homologous transfusions and the overall red cell units of ABT among treatment groups. Thus, PAT in total hip or knee arthroplasty was found to be an effective way to significantly reduce the need for ABT.

Many potential factors such as tourniquet use, hypertension, cement, and surgical skill can lead to a difference in blood loss after joint surgery. The use of a tourniquet was found to decrease intra-operative blood loss but could not influence the postoperative blood loss in drains or affect transfusion rates [34]. Hypertension may lead to increased blood loss and increased transfusion requirements. However, no differences were found in the total calculated blood loss between patients with or without hypertension [17]. In total hip replacement surgery, a significant portion of blood loss occurred during the intra-operative period, and there was a general consensus with regard to the nature of intra-operative blood loss [23], [30]. Our meta-analysis found less blood loss in the PAT group than in the control group (MDs, −131.10; 95% CIs, −257.13 to −5.07; P = 0.04) (Figure 4). The exact mechanism underlying the reduced blood loss in PAT patients has yet to be determined, but it seems that blood loss can be reduced by preventing the use of banked blood [29], [35]–[37]. It is therefore clearly preferable not to transfuse ABT if possible, especially in the context of orthopedic surgery.

The point at which to administer a blood transfusion is controversial. In the six studies included, the administration of banked blood was determined by the Hb value and/or clinical signs (e.g., blood pressure, pulse). The observed values were typical of those reported elsewhere. Recently, physicians have agreed that Hb value is a strong trigger for transfusion and that the clinical symptoms of anemia should always be considered when contemplating transfusion [35]. However, our understanding of human physiology, oxygen delivery and the safety and risks of ABT has changed considerably. Therefore, it was also suggested that decisions about transfusions should be based on an assessment of the patient’s clinical needs and symptoms rather than laboratory values alone [38].

One of the trials included observed that 7 of the 13 patients in the study group that required ABT received it after day 5, when the Hb eventually fell below 9 g/dl [12]. In the control group, only one patient required ABT on day 5, whereas all of the others required ABT on day 2 after surgery. This effect was caused by a higher Hb level in the patients of the PAT group on day 2 (MDs, 0.45; 95% CIs, 0.11 to 0.80; P = 0.01) than in patients of the control group, which suggests that PAT prevents a rapid decrease in the Hb level during the early post-operative period. However, by day 5, this benefit disappeared (MDs, 0.29; 95% CIs, −0.01 to 0.59; P = 0.06). This meta-analysis showed that PAT was effective in reducing ABT but not useful in achieving high postoperative Hb levels. These findings suggested that PAT was not useful in achieving high postoperative Hb levels to enhance recovery, which agreed with the findings of Adrianus [15].

The pooled results indicated significant differences between treatment groups with regard to the rate of febrile reactions (RRs, 0.65; 95% CIs, 0.46 to 0.93; P = 0.02) (Figure 6). The rate (30%) of mild febrile transfusion reactions was higher in Soosman’s study [3] than in previous reports [39], which may have been the result of the inclusion of all mild febrile reactions (>1°C increase in temperature). A lower rate of re-infusion may prevent febrile reactions. It has been suggested that this rise in temperature was a response to the surgical procedure itself [25]. Therefore, postoperative febrile reactions are generally seen in the context of major orthopedic surgery. Some studies have indicated a lack of a difference in developing febrile reactions when using the PAT system or a regular postoperative low-vacuum drainage system. Nearly the same extent of patients with febrile reactions was found in both groups (RRs, 0.88; 95% CIs, 0.46 to 1.67; P = 0.69) [40], [41]. Pooling these results with regard to the incidence of febrile reactions showed that adverse effects (chills and febricula) developed during PAT in some patients but less often than during ABT, which agreed with previous reports [18], [23], [25]. The exact mechanism underlying the higher rates of febrile episodes after ABT has yet to be determined, but it seems that this problem could be reduced by not using ABT [19].

Some reports [2], [42], [43] showed that the postoperative infection rate was significantly lower in PAT than in ABT patients. del Trujillo et al [17] observed no adverse effect of PAT return during the study but observed a tendency for a reduced rate of postoperative infections in patients receiving PAT. In contrast, an increased post-operative rate of infection was found in ABT patients [2]. The exact mechanism for this effect is not known; however, various theories exist, including one based on the immuno-depressant effects of ABT [44]. The observed effects also may be related to the immunostimulatory and anti-inflammatory effects of PAT [17], [19]. Our meta-analysis finding indicated similar rates of infection in patients that received PAT compared with those that received ABT. However, because of the low incidence of infection, this systematic analysis was not sufficiently powered to compare the treatment groups with respect to postoperative infection rate. It is clearly preferable not to transfuse blood if possible, especially in the context of orthopedic surgery, in which deep infection is a devastating complication.

In a previous multivariate analysis, the re-infusion group remained an independent variable in LOHS. A reduction in LOHS was observed by Newman and Shulman [19], [45], who found a reduction of 2 days, but this reduction was associated with a reduction in allogeneic RBCs. Newman also postulated that the use of a reinfusion technique after total knee arthroplasty could shorten the hospital stay by rendering the patients less febrile and infective [46]. Similar observations were made in other studies, in which each ABT-transfused patient stayed in the hospital for one additional day [35], [47]. Similarly, the use of PAT blood after total knee arthroplasty appeared to decrease the LOHS and effectively reduce the postoperative requirements for ABT, as has been confirmed by recent studies [4], [48]. It has recently been reported that hospitalization costs more for patients who undergo joint replacement when ABT is used. Therefore, the use of a PAT technique after total joint replacement can reduce costs by decreasing the ABT and decreasing the LOHS. This reduction in the hospital stay, which is probably related to decreased anxiety about the possibility of infection, is welcome by the hospital staff and patients. As a result, PAT following total joint arthroplasty not only reduces the need for ABT but also reduces the cost to the patient, as it is an inexpensive method. However, any reduction in the LOHS in patients receiving PAT versus ABT is difficult to evaluate, because without rigid criteria for discharge, standards may change slightly during the study period. Therefore, this connection between LOHS and cost should be interpreted cautiously because our study was not powered for this conclusion.

### Conclusion

The use of PAT effectively reduces the demand for homologous banked blood transfusions in patients who have undergone total knee or hip arthroplasty. The LOHS and post-operative febrile reaction rate in the PAT groups were slightly reduced compared with the levels in the control group. Therefore, we would like to emphasize that the main benefit of this system was the ability to reduce banked blood utilization without compromising patient safety. We believe that in operations such as total knee arthroplasty, where a tourniquet is used intra-operatively and collection drains are used postoperatively and almost universally, PAT is the most appropriate method of autologous blood transfusion. The meta-analysis results suggest that there are clinical and economical advantages in avoiding the use of ABT blood. We therefore strongly recommend that PAT become more widely used, especially in operations such as total knee or hip arthroplasty where substantial blood loss is anticipated. Therefore, we conclude that PAT in total knee or hip arthroplasty is a safe and effective way to decrease ABT.

To our knowledge, this is the first meta-analysis to systematically compare the clinical results of PAT (postoperative auto-transfusion) with a control treatment in a population of patients undergoing total knee or hip arthroplasty who did not donate autologous blood pre-operatively. We found that the use of a PAT system significantly decreased the risk of ABT and LOHS. The use of PAT could also reduce the occurrence of wound complications and febrile reactions. This study, together with other previously published data, suggests that PAT drains are beneficial. A much larger study is required to confirm these results.

In summary, this meta-analysis has proven that the use of a PAT reinfusion system reduced significantly the demand for ABT, the number of patients who require ABT and the cost of hospitalization after total knee and hip arthroplasty. Larger, sufficiently powered studies are necessary to evaluate the presumed reduction in the incidence of infection as well as DVT after joint arthroplasty with the use of PAT.

## Supporting Information

Checklist S1 (DOC)
