# Supplementary material for: Post-Operative Auto-Transfusion in Total Hip or Knee Arthroplasty: A Meta-Analysis of Randomized Controlled Trials
Source: PLoS One. 2013 Jan 25;8(1):e55073. doi: 10.1371/journal.pone.0055073 (PMC3555861; doi:10.1371/journal.pone.0055073)
Supplement: Checklist S1 — (DOC) [file pone.0055073.s001.doc]

| **Section/topic** | **#** | **Checklist item** | **Reported on page #** |
| --- | --- | --- | --- |
| **TITLE** | | |  |
| Title | 1 | Post-operative Auto-Transfusion in Total Hip or Knee Arthroplasty: Meta-Analysis from Randomized Controlled Trials | Title |
| **ABSTRACT** | | |  |
| Structured summary | 2 | Background: Total hip or knee arthroplasty is an elective procedure usually accompanied by substantial blood loss, which may lead to acute anemia. As a result, almost half of total joint arthroplasty patients receive allogeneic blood transfusion (ABT). Many studies have shown that post-operative auto-transfusion (PAT) reduces significantly the need for ABT, but others have questioned the efficacy of this method.  Objectives: To evaluate the efficacy of PAT, we conducted a Cochrane systematic review that combined all available data from randomized controlled trials(RCTs).  Data sources: Studies published in English in The Cochrane Library (Issue 2, 2011), PubMed (January 1995 to February 2011), Ovid (January 1995 to February 2011), ScienceDirect Online (January 1995 to February 2011), ISI Web of Knowledge (January 1995 to February 2011), several orthopedic journals, transfusion journals, conference proceedings.  Study eligibility criteria, participants, and interventions: patients undergoing total knee or hip arthroplasty，without considering primary or revision of the replacement. Patients were excluded from the trials if they had received any other blood-saving strategy, such as preoperative autologous blood donation, erythropoietin treatment, iron supplementation and so on.  Study appraisal and synthesis methods: The datas were pooled using REVMAN 5.0 software (The Nordic Cochrane Centre, Copenhagen, Denmark). For each study, we calculated relative risks (RRs) with 95% confidence intervals (CIs) for dichotomous datas and Mean Differences (MDs) with 95% CIs for continuous datas.  Results: We found that PAT significantly decreased the demands of ABT and length of hospital stay, in comparison with control.  Conclusions and implications of key findings: To our knowledge, this is the first meta-analysis to compare the clinical results between PAT and the control, in joint replacement patients. | Structured summary |
| **INTRODUCTION** | | |  |
| Rationale | 3 | Total hip or knee arthroplasty is usually accompanied by substantial blood loss, which may lead to acute anemia. As a result, most of total joint arthroplasty patients receive allogeneic blood transfusion (ABT) to avoid the risk of postoperative anemia. Many studies have shown that PAT reduces significantly the need for ABT, but others have questioned the efficacy of this methodor found that PAT after arthroplasty had a limited blood saving effect. | Objectives |
| Objectives | 4 | The main objective of this study was to compare the clinical results of post-operative auto-transfusion (PAT) with the control group (receive standardized drainage，if necessary patients are given allogeneic blood transfusion) following total hip or knee arthroplasty. | Objectives |
| **METHODS** | | |  |
| Protocol and registration | 5 | The data were extracted by 2 reviewers independently to ensure accuracy. In cases of disagreement, consensus was reached by discussion. Study quality was evaluated according to the method for RCTs described in the Cochrane Reviewer's Handbook 5.0.  No registration information and registration number. | Inclusion and Exclusion Criteria |
| Eligibility criteria | 6 | Study characteristics: the numbers of patients need at least one unit of allogeneic RBCs; ABT index (units of RBC per patient transfused); volume of blood loss post-operation; the post-operative Hb level; febrile reaction frequencies; infection rate  The published results of relevant trials from anuary 1995 to February 2011 in English. | Inclusion and Exclusion Criteria |
| Information sources | 7 | We searched for the published results of relevant trials in The Cochrane Library (Issue 2, 2011), PubMed (January 1995 to February 2011), Ovid (January 1995 to February 2011), ScienceDirect Online (January 1995 to February 2011), ISI Web of Knowledge (January 1995 to February 2011), several orthopedic journals, transfusion journals, conference proceedings. When necessary, the authors of the articles were contacted for original information.  Date last searched: 31^st^ , March 2011. | Literature Search |
| Search | 8 | Search strategy: MEDLINE (OVID)   1. total hip replacement/ 2. total hip arthroplasty/ 3. total knee replacement/ 4. total knee arthroplasty/ 5. total joint arthroplasty/ 6. 1 or 2 or 3 or 4 or 5 7. autologous transfusion/ 8. post-operative blood salvage/ 9. post-operative blood salvage/ 10. 7 or 8 or 9 11. 6 and 10 12. randomized controlled trials/ 13. controlled-clinical-trial/ 14. random allocation/ 15. double-blind method/ 16. single-blind method/ 17. 12 or 13 or 14 or 15 or 16 18. 11 and 17 | Literature Search |
| Study selection | 9 | Fisrt, browsing the abstract of these records after duplicates removed, then screening the 270 records included. Second, excluding 245 full-text articles, with reasons: 1, not RCTs. 2,Not suitable for this analysis for no enough information. There was 25 full-text articles assessed for eligibility.  Third, 8 studies included in quantitative synthesis for meta-analysis. | See flow diagram |
| Data collection process | 10 | We developed a data extraction sheet (Cochrane Consumers and Communication Review Group’s data extraction template), pilot-tested it on 6 randomly-selected included studies, and refined it accordingly.The data were extracted by 2 reviewers independently to ensure accuracy. In cases of disagreement, consensus was reached by discussion of all investigators. | Inclusion and Exclusion Criteria |
| Data items | 11 | Participants: patients undergoing total knee or hip arthroplasty,without considering primary or revision of the replacement.  Interventions: post-operative auto-transfusion (PAT) or allogeneic blood transfusion (ABT).  Comparisons: the patients undergoing PAT and the patients undergoing ABT.  Outcomes: The primary outcome measures include the number of patients need at least one unit of allogeneic RBCs(patients requiring homologous blood transfusions)and ABT index (units of RBC per patient transfused). The secondary outcome measures include total volume of blood loss following surgery, postoperative hemoglobin(Hb) level, transfusion reactions, infections, deep vein thrombosis (DVT), as well as length of hospital stay (LOHS).  Study design: present protocol for the transfusion of the total joint arthroplasty.  Funding sources: no funding sources. | Outcome Measures |
| Risk of bias in individual studies | 12 | To ascertain the validity of eligible randomized trials, pairs of reviewers working independently and with adequate reliability determined the adequacy of randomization and concealment of allocation, blinding of patients, health care providers, data collectors, and outcome assessors. | Literature Search |
| Summary measures | 13 | The principal summary measures: For each study, we calculated relative risks (RRs) with 95% confidence intervals (CIs) for dichotomous datas and Mean Differences (MDs) with 95% CIs for continuous datas. | Statistical Analysis |
| Synthesis of results | 14 | we pooled the results of comparable groups of trials using both the fixed effects (Mantel-Haenszel test) and random effects (DerSimonian-Laird method) models. A random effects model was used when significant heterogeneity was detected between studies (P<.10; I^2^>50%). | Statistical Analysis |

| Risk of bias across studies | 15 | For each trial we plotted the effect by the inverse of its standard error. The symmetry of such ‘funnel plots’ was assessed both visually, and formally with Egger’s test, to see if the effect decreased with increasing sample size. | Statistical Analysis |
| --- | --- | --- | --- |
| Additional analyses | 16 | no |  |
| **RESULTS** | | |  |
| Study selection | 17 | 270 studies screened, 25 full-text articles assessed for eligibility, and 6 articles assessed included in the meta-analysis. Reasons for exclusions at each stage, seen in the flow diagram. | See flow diagram |
| Study characteristics | 18 | All 6 studies finally selected for the analysis were randomised controlled trials published in English.  The included studies involved 829 participants, 469 in PAT group and 360 in ABT group. | Literature Search |
| Risk of bias within studies | 19 | Present data on risk of bias of each study and, if available, any outcome level assessment (see item 12). |  |
| Results of individual studies | 20 | See forest plots in Figure 2-8. | Figure 2-8 |
| Synthesis of results | 21 | 1, The number of patients need at least one unit of allogeneic RBCs(patients requiring homologous blood transfusions): (RRs, 0.52; 95% CI, 0.32 to 0.85; P = .009).  2, ABT index (units of RBC per patient transfused): (MDs, -0.68; 95% CI, -1.33 to -0.04; P =0.04).  3. the volume of the blood loss: (MDs, -73.89; 95% CI, -112.79 to -34.99; P =0.0002).  4. The post-operation Hb level: (MDs, 0.21; 95% CI, 0.09 to 0.34; P =0.005)  5. febrile reaction frequencies: (RRs, 0.65; 95% CI, 0.46 to 0.93; P = .02).  6. infection rate: (RRs, 0.83; 95% CI, 0.44 to 1.57; P = .44). | Outcome Measures |
| Risk of bias across studies | 22 | To explore the heterogeneity, a funnel plot was drawn. The funnel plot shows evidence of considerable asymmetry. | Outcome Measures |
| Additional analysis | 23 | no |  |
| **DISCUSSION** | | |  |
| Summary of evidence | 24 | PAT in total hip or knee arthroplasty was an effective way to reduce significantly the need for ABT.  Our meta-analysis showed less blood loss in the PAT group than in the control group. It seems that blood loss can be reduced by avoiding the use of banked blood.  This meta-analysis showed that the PAT was effective in reducing ABT but not useful in trying to reach high postoperative Hb levels.  PAT following total joint arthroplasty not only reduces the need for ABT, but also reduces the cost as it is an inexpensive method.  It is therefore clearly preferable not to transfuse ABT if possible, especially in orthopedics surgery. | discussion |
| Limitations | 25 | The main limitation of this meta-analysis, is that the patient population and the outcome definitions are not the same across studies. Publication bias might account for some of the effect we observed. Smaller trials are analyzed with less methodological rigor than larger studies, and an asymmetrical funnel plot suggests that selective reporting may have led to an overestimation of effect sizes in small trials.  A further much larger study is required to define if there is any benefit from an PAT over no PAT at all. | discussion |
| Conclusions | 26 | The use of PAT reinfusion system was highly efficient to reduces the demands of ABT, the number of patients who require ABT and the cost of hospitalization after total knee and hip arthroplasty.  Larger sufficiently powered studies are necessary to evaluate presumed reduction in incidence of infection as well as DVT after joint arthroplasty by the use of PAT. | Conclusions |
| **FUNDING** | | |  |
| Funding | 27 | No current external funding sources for this study. |  |
